# Differential Intracochlear Sound Pressure Measurements in Human Temporal Bones with an Off-the-Shelf Sensor

**DOI:** 10.1155/2016/6059479

**Published:** 2016-08-16

**Authors:** Martin Grossöhmichen, Rolf Salcher, Klaus Püschel, Thomas Lenarz, Hannes Maier

**Affiliations:** ^1^Cluster of Excellence Hearing4all, Germany; ^2^Department of Otolaryngology and Institute of Audioneurotechnology (VIANNA), Hannover Medical School, 30625 Hannover, Germany; ^3^Department of Legal Medicine, University Medical Center Hamburg-Eppendorf, 22529 Hamburg, Germany

## Abstract

The standard method to determine the output level of acoustic and mechanical stimulation to the inner ear is measurement of vibration response of the stapes in human cadaveric temporal bones (TBs) by laser Doppler vibrometry. However, this method is reliable only if the intact ossicular chain is stimulated. For other stimulation modes an alternative method is needed. The differential intracochlear sound pressure between scala vestibuli (SV) and scala tympani (ST) is assumed to correlate with excitation. Using a custom-made pressure sensor it has been successfully measured and used to determine the output level of acoustic and mechanical stimulation. To make this method generally accessible, an off-the-shelf pressure sensor (Samba Preclin 420 LP, Samba Sensors) was tested here for intracochlear sound pressure measurements. During acoustic stimulation, intracochlear sound pressures were simultaneously measurable in SV and ST between 0.1 and 8 kHz with sufficient signal-to-noise ratios with this sensor. The pressure differences were comparable to results obtained with custom-made sensors. Our results demonstrated that the pressure sensor Samba Preclin 420 LP is usable for measurements of intracochlear sound pressures in SV and ST and for the determination of differential intracochlear sound pressures.

## 1. Introduction

The majority of Implantable Middle Ear Hearing Devices (IMEHDs) such as the MET® (Cochlear Ltd.) or the Vibrant Soundbridge (MED-EL) were initially developed for the treatment of sensorineural hearing loss [1, 2]. For this purpose, the implant converts external sound to vibration, which mechanically drives the ossicular chain (e.g., incus body).

To quantify the IMEHD's equivalent sound pressure output level in such applications, the ASTM standard 2504-05 [3] provides an experimental method in human cadaveric temporal bones (TBs). This method is commonly used and is based on the comparison of the vibration amplitude of the stapes footplate (SFP) in response to sound and to actuator stimulation measured with a laser Doppler vibrometer (LDV). This method has been demonstrated to be reliable for applications that stimulate the ear in the physiological forward direction [2, 4–6].

More recently, the indication of IMEHDs was extended to conductive and mixed hearing loss applications where the implant vibrates the SFP or the round window (RW) [7–9]. Likewise, the direct acoustic stimulation of the cochlea by a Direct Acoustic Cochlear Implant (DACI) such as the Codacs*™* (Cochlear Ltd.) has become well established for the treatment of severe to profound mixed hearing loss [10, 11]. However, quantifying the output level of these new stimulation modes by LDV measurements in TBs according to ASTM standard 2504-05 [3] is not possible.

In the case where the SFP is obscured, vibration responses of the RW of the cochlea can be determined by LDV instead [12, 13]. However, due to the complex vibration pattern of the RW at frequencies >1.5 kHz, this method is reliable only within one experiment and if the measurement site on the RW is unchanged [12, 14, 15]. In the other case, where the RW is excited by an IMEHD, the ear is stimulated in reverse direction compared to the physiological sound transmission. Although SFP vibration responses are commonly measured to estimate the stimulation efficiency in such cases [16–18], it has been demonstrated that this method markedly underestimates the real output level in reverse stimulation [6]. In direct acoustic stimulation by a DACI the SFP is perforated and the cochlear fluids are stimulated by a piston. However, opening the cochlea causes strong changes in the motion pattern of the RW vibration response at frequencies >1.5 kHz making vibration measurements unreliable [15].

In conclusion, measuring vibration responses according to ASTM standard 2504-05 [3] is a reliable method to determine the output level of IMEHDs only during forward stimulation and only if the ossicular chain and cochlea are left intact. In all other stimulation modes an alternative measurement method is needed to quantify the output level of IMEHDs and DACIs in human cadaveric TBs. Measuring the sound pressure difference between the two compartments of the cochlea, scala vestibuli (SV) and scala tympani (ST), represents such a method as the pressure difference correlates with cochlear excitation [19]. Pressure differences have successfully been used to characterize the output level in forward and reverse stimulation in TB experiments [6, 20–22]. However, in these studies the sound pressure in SV and ST was measured with a custom-made pressure sensor developed by Olson [23]. This sensor is commercially not available and difficult to build. Therefore, the much-needed technique of intracochlear sound pressure measurement is currently available only for a limited group of researchers.

In order to make this method accessible to a wider community, the goal of our study was to demonstrate that an off-the-shelf pressure measurement system can be successfully used for intracochlear sound pressure measurement. This may contribute to establishing intracochlear sound pressure measurements as a generally accessible and commonly used technique and a strong tool in hearing research beside vibration measurements with LDVs.

## 2. Materials and Methods

In this study intracochlear sound pressures in response to acoustic stimulation of the tympanic membrane were measured in SV (*P*_SV_) and ST (*P*_ST_) in cadaveric human TBs with the off-the-shelf pressure transducer Samba Preclin 420 LP.

### 2.1. TB Preparation

Nineteen human cadaveric TBs obtained from the Institute for Pathology of the Hannover Medical School and the Department of Legal Medicine of the University Medical Center Hamburg-Eppendorf [24] were used for this study. Harvesting and anonymous use of the TBs were approved by the ethics committee of the Hannover Medical School (2168-2014). All TBs were harvested within 48 h* postmortem*, immediately frozen at approximately −19°C, and thawed shortly before preparation at room temperature. A mastoidectomy, removal of the facial nerve, and thinning of the RW niche overhang down to approximately 0.5–1 mm were performed. This wide mastoid preparation exposed the SFP and the promontory where the pressure transducers were intended to be inserted. After preparation the TBs were stored in saline containing ~0.005‰ thimerosal at approximately −19°C until the experiments. In the beginning of each experiment the integrity of the RW membrane and the mobility of the ossicular chain were carefully checked using a surgical microscope (OPMI-1, Zeiss, Germany) and surgical tools. During experiment the TBs were kept moist with saline to avoid changes in mechanical behavior [3].

### 2.2. Experimental Setup

TBs were fixed in a laboratory clamp on a vibration isolated table (LW3048B, Newport, Germany). A custom-made sound application setup containing a probe microphone (ER-7C, Etymotic Research Inc., USA) and a loudspeaker (DT48, Beyerdynamic, Germany) was cemented (Paladur, Heraeus Kulzer GmbH, Germany) in the outer ear canal (OEC). The tip of the microphone's probe tube was positioned 1-2 mm from the tympanic membrane (TM). To prevent unwanted vibrations from being transferred from the loudspeaker to the TB, the TB was embedded in modelling clay (Play-Doh, Hasbro, Germany).

### 2.3. Intracochlear Pressure Measurement

Intracochlear pressures in SV and ST were measured simultaneously with two off-the-shelf pressure fiber-optic transducers (Samba Preclin 420 LP, Samba Sensors AB, Sweden) connected to a two-channel control unit (Samba control unit 202, Samba Sensors AB, Sweden). The pressure transducer is based on the Fabry-Pérot interferometer principle, has an outer diameter of 0.42 mm, is calibrated by the manufacturer, valid for lifetime with a long term stability of <0.5% of range [25, 26], and can be reused for several measurements. In this study three sensors were used as the membrane of one sensor was damaged during experiments and two sensors are needed in each experiment for the simultaneous measurement. The pressure measurement system has a maximum measurement frequency of 40 kHz, a pressure range of −50 to +350 hPa and a sensitivity of approximately −80 dB re 1 V/Pa. Under the assumption of a middle ear amplification of 23 dB at ≤1 kHz, 0 dB at ≥7 kHz, and a decrease of −8.6 dB/octave in between [27] and depending on the conversion range of the AD/DA converter the theoretical resolution limit of the samba pressure measurement system is between 65 dB SPL and 72 dB SPL input to the TM at ≤1 kHz and between 88 dB SPL and 95 dB SPL at ≥7 kHz. The control unit provides a proportional voltage signal at each analog output channel. Each transducer was mounted to a custom-made holder attached to a 3-axis micromanipulator (M3301R, World Precision Instruments Germany GmbH, Germany), allowing the adjustment in all three spatial directions.

### 2.4. Vibration Measurement

Stapes footplate (SFP) vibration responses were measured with a single-point LDV system (OFV 534, OFV 5000, A HLV MM 30, Polytec, Germany) attached to a surgical microscope (OPMI-1, Zeiss, Germany). The laser beam was directed at a small piece (<0.3 mm × 0.3 mm) of reflective tape on the SFP at a visually estimated incident angle of ≤45° to the SFP normal. During analysis the incident angle was considered by a cosine correction.

### 2.5. Experimental Procedure

First, the TB preparation was checked visually using the surgical microscope. In case of damage such as a ruptured RW or broken SFP the TB was rejected.

Second, the loudspeaker was driven by a custom multisine signal, having equal amplitudes of approximately −25 dB re 1*V*_rms_ at 0.125, 0.25, 0.5, 1, 2, 3, 4, 6, 8, and 10 kHz. Simultaneously the vibration of the SFP was measured with the LDV and the sound pressure level (SPL) in the OEC was recorded by the probe microphone. Only if the SFP vibration response was within the modified acceptance range [4] of the ASTM standard F2504-05 [3], the experiment was continued.

**Figure 1:**
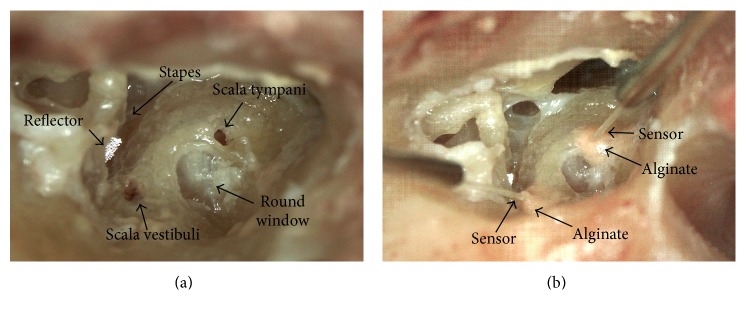
Temporal bone preparation for intracochlear sound pressure measurements. (a) Cochleostomies of ~0.5 mm diameter in scala vestibuli (SV) and scala tympani (ST) (picture was taken after the experiment). The reflector was placed on the stapes footplate for LDV measurement. (b) Samba Preclin 420 LP transducers placed in SV (left) and ST (right), sealed with alginate.

Third, two Samba Preclin 420 LP transducers were placed in SV and ST (Figure 1). For this purpose the promontory was first thinned where the cochleostomies were intended and then a fenestration of approximately 0.5 mm diameter was made in SV and ST using a diamond burr and a footplate perforator. When the tip of the transducer was inserted 100–300 *μ*m (visually estimated) into the scalae, the transducer was sealed with the surrounding bone using dental impression material alginate (Alginoplast®, Heraeus Kulzer GmbH) in TB05–07 or a silicone rubber plug (Silikonkautschuk RTV, Wacker-Chemie GmbH, Germany) permanently mounted to the optical fiber in TB16, 18, and 19. If necessary, the silicone rubber could be easily removed by pulling it off the fiber. During cochleostomy, sensor insertion, and sealing, the middle ear cavity was immersed in saline to prevent air from entering the cochlear.

Fourth, SFP vibrations were measured in response to the acoustic multisine stimulation similar to the second step of the experiment. With this measurement the effect of the cochleostomy and sensor insertion on the SFP vibration responses to sound stimulation was investigated.

Fifth, the TM was stimulated acoustically between 0.1 and 10 kHz by a sequence of sine-wave signals with a frequency resolution of 3/octave (resulting in 23 frequencies between 0.1 and 10 kHz) at levels of 105–130 dB SPL. Simultaneously the sound pressures in SV and ST were measured by the pressure transducers, the SPL at the TM by the probe microphone and the vibration of the SFP by the LDV. Finally for the analysis single frequency results of the 23 stimulation frequencies were assembled into one frequency response for each TB.

Sixth, after completing all measurements the pressure transducers were removed and the correct positioning of the cochleostomies in SV and ST was confirmed visually by dissection of the TB.

In total, three out of 19 TBs were excluded due to damage of anatomical structures. Of the remaining 16 TBs, six had SFP vibration responses compliant to the modified acceptance range of ASTM F2504-05 [4] and were used for intracochlear sound pressure measurements.

### 2.6. Signal Generation, Acquisition, and Analysis

For signal generation and acquisition a commercial 16-bit, 4-channel data acquisition system (PC-D and VIB-E-400, Polytec, Germany) with commercial software (VibSoft 4.8.1, Polytec, Germany) was used. Electric input signals to the loudspeaker were buffered by an amplifier (SA1, Tucker-Davis Technologies, USA). Electric output signals from probe microphone, LDV, and Samba pressure measurement system were acquired simultaneously as averaged complex spectra using 800 (Fast Fourier Transformation) FFT lines between 0 and 10 kHz with 12.5 Hz resolution. The signal-spectra obtained during multisine stimulation in the 2nd and 4th steps of the experiment were averaged 500 times and the signal-spectra obtained during stimulation with the sequence of sine-wave signals (5th step of experiment) were averaged 1000 times to increase the signal-to-noise ratio (SNR). At each stimulation frequency the SNR was determined using the average of the three adjacent FFT lines below and above as noise level estimate. Vibration responses with SNR < 12 dB and intracochlear sound pressure responses with SNR < 7 dB were excluded from analysis. Empirically these values have proved to be sufficient to record signals clearly above the noise floor. The differential sound pressure across the cochlear partition (Δ*P* = *P*_SV_ − *P*_ST_) was calculated by subtracting the complex pressures in ST (*P*_ST_) and SV (*P*_SV_) in the frequency domain.

## 3. Results

### 3.1. SFP Vibration Responses before and after Cochleostomy

**Figure 2:**
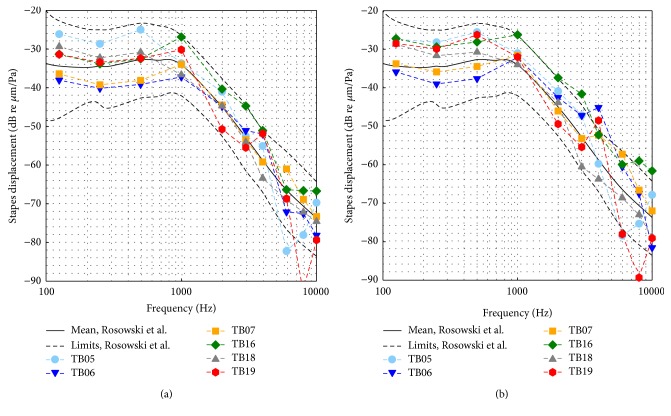
SFP responses to sound stimulation at the tympanic membrane in TB preparations used for analysis. (a) Before pressure sensor insertion. (b) After cochleostomy and pressure sensor insertion. The black dashed lines depict the limits given by Rosowski et al. [4].

Six TBs showed vibration responses of the SFP to sound [dB re *μ*m/Pa] at 0.25–4 kHz (Figure 2(a)) compliant to the modified acceptance range [4] of ASTM F2504-05 [3] and were used for intracochlear sound pressure measurements. Even after the insertion of the pressure transducer, the SFP responses (Figure 2(b)) were inside the range, except TB06 at 4 kHz (5.7 dB deviation), TB16 at 1, 2, and 3 kHz (2.8 dB maximum deviation), and TB19 at 4 kHz (2.4 dB deviation).

### 3.2. Sound Pressures in Scala Vestibuli and Scala Tympani

**Figure 3:**
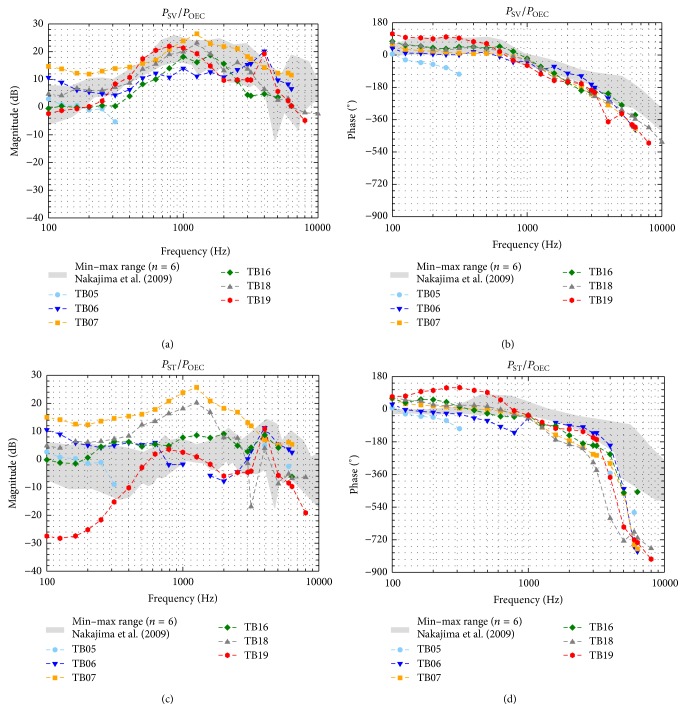
Sound pressures in scala vestibuli (*P*_SV_, (a) and (b)) and scala tympani (*P*_ST_, (c) and (d)) normalized to the outer ear canal sound pressure level (*P*_OEC_). For comparison the range of results obtained with a custom-made pressure sensor by Nakajima et al. [21] is given (grey shaded area). Data with an SNR < 7 dB were omitted.

**Figure 4:**
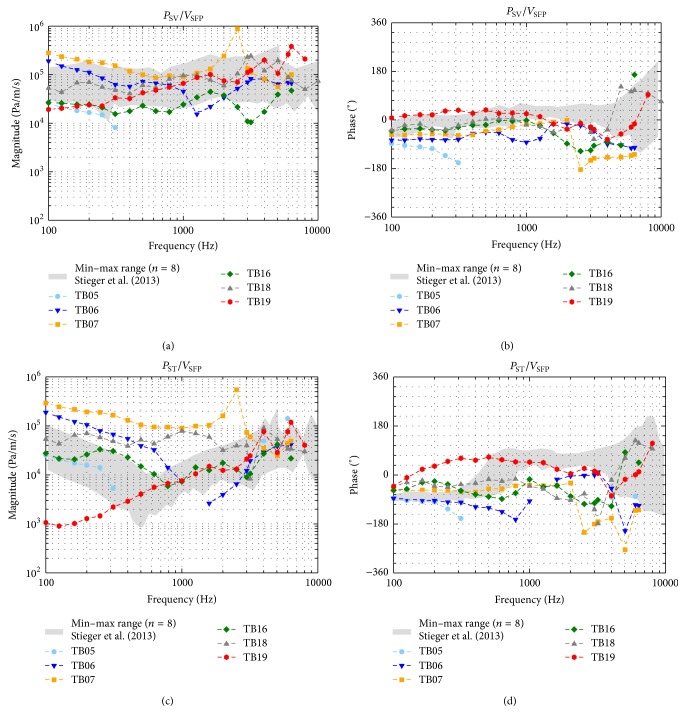
Sound pressures in scala vestibuli (*P*_SV_, (a) and (b)) and scala tympani (*P*_ST_, (c) and (d)) normalized to the stapes footplate velocity (*V*_SFP_). For comparison the range of results obtained with a custom-made pressure sensor by Stieger et al. [6] is given (grey shaded area). Data with an SNR < 7 dB were omitted.

**Figure 5:**
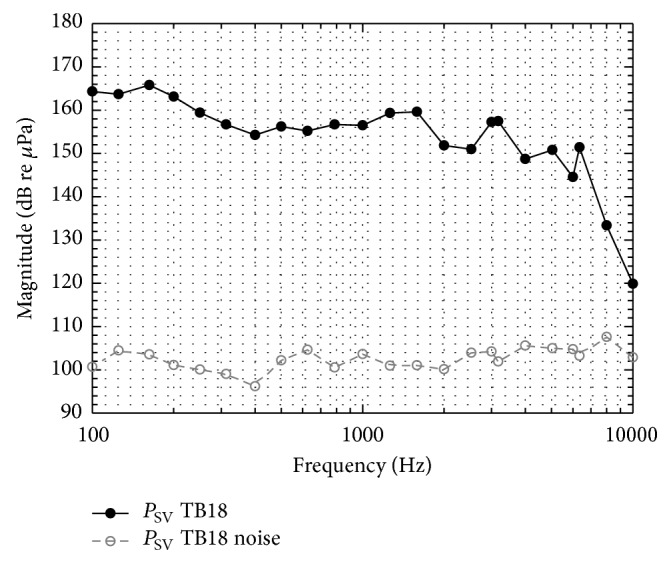
An example (TB18) of the sound pressure Psv measured in scala vestibuli and the corresponding noise floor estimated by the average of the three adjacent FFT lines below and above each stimulations frequency.

To compare the measurement data across all TBs independent of stimulation level, the intracochlear sound pressures *P*_SV_ and *P*_ST_ were normalized to the outer ear canal SPL *P*_OEC_ (Figure 3) and to the stapes footplate velocity *V*_SFP_ (Figure 4). In all specimens except TB05, intracochlear sound pressures were measurable in both scalae between 0.1 and 6.35 kHz with an SNR > 7 dB. Pressures at 8 kHz were measurable in two experiments (TB18, TB19) and at 10 kHz in one experiment (TB18, Figure 5). When normalized to *P*_OEC_ the magnitudes of *P*_SV_ (Figure 3(a)) were similar across all experiments, as well as *P*_ST_ (Figure 3(c)) at frequencies ≥3 kHz. At lower frequencies the magnitudes of *P*_ST_/*P*_OEC_ varied up to 42 dB across experiments. In particular, in TB19 the magnitudes were at ≤0.4 kHz up to 27 dB smaller than in all other experiments. The phases of *P*_SV_ (Figure 3(b)) and *P*_ST_ (Figure 3(d)) were similar across all TBs showing an increasing lag to *P*_OEC_ with increasing frequency. At frequencies >4 kHz the phases of *P*_ST_ decreased significantly, resulting in approximately two cycles shift at ≥5.5 kHz. The magnitudes of *P*_SV_ normalized to *V*_SFP_ (Figure 4(a)) were similar across all experiments; only TB07 showed a prominent peak at 2.525 kHz. In contrast, the magnitudes of *P*_ST_/*V*_SFP_ (Figure 4(c)) varied at frequencies below 3 kHz significantly by up to 49 dB. Again, at frequencies ≤0.4 kHz the magnitudes in TB19 were distinctly smaller compared to the other experiments. At frequencies ≤2 kHz the *P*_SV_/*V*_SFP_ and *P*_ST_/*V*_SFP_ phases were mainly constant for each TB whereas at higher frequencies the phases showed a higher variation.

In each experiment the normalized magnitude of *P*_SV_ was higher than the normalized magnitude of *P*_ST_ at frequencies above 400 Hz whereas the pressure magnitudes in both scalae were similar at lower frequencies. Only in TB07 the magnitudes of *P*_SV_ and *P*_ST_ were similar (differences ≤ 2 dB) up to 1.6 kHz and in TB19 the magnitude of *P*_SV_ was distinctly higher than *P*_ST_ at all frequencies.

### 3.3. Intracochlear Pressure Differences

**Figure 6:**
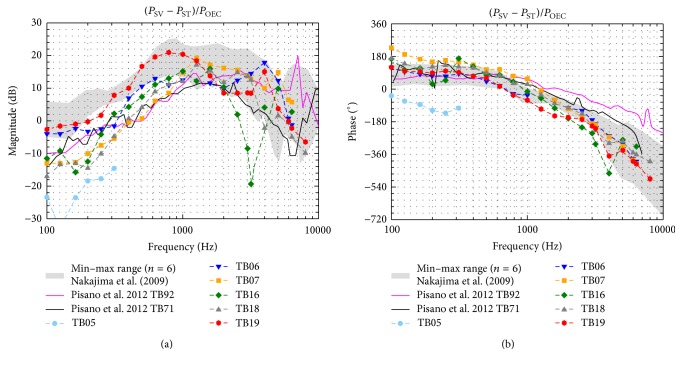
Pressure differences (*P*_SV_ − *P*_ST_) normalized to the outer ear canal sound pressure level (*P*_OEC_). For comparison the range of results (Nakajima et al. [21], grey shaded area) and two exemplary measurements (Pisano et al. [22], solid lines) obtained with a custom made pressure sensor are given. Data with an SNR < 7 dB were omitted.

**Figure 7:**
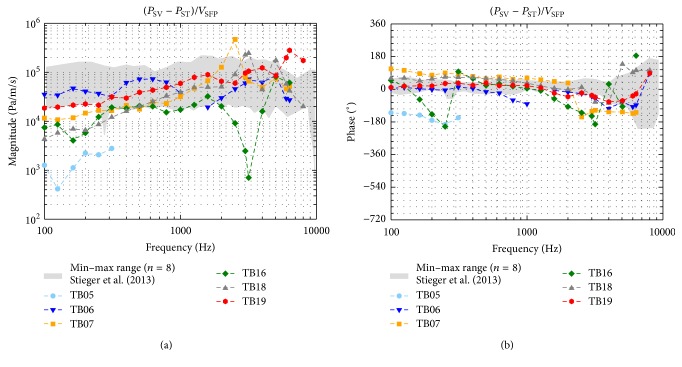
Pressure differences (*P*_SV_ − *P*_ST_) normalized to the SFP velocity (*V*_SFP_). For comparison the range of results obtained with a custom made pressure sensor by Stieger et al. [6] is given (grey shaded area). Data with an SNR < 7 dB were omitted.

The magnitudes and phases of the complex pressure differences (Δ*P* = *P*_SV_ − *P*_ST_) between SV and ST are plotted in Figure 6 normalized to the SPL in the OEC (*P*_OEC_). Apart from TB16, showing a sharp notch at 2525–3175 Hz, the magnitudes (Figure 6(a)) were similar across all TBs with differences ≤ 20 dB. The phases (Figure 6(b)) were also similar in all TBs showing a 1/8–2/3 cycle lead at frequencies below 1 kHz that decreased with increasing frequency to a lag of up to 1 1/3 cycle. Since in TB05 pressure differences were only measurable at ≤312.5 Hz and up to 20 dB lower than in the other experiments, it was assumed that the preparation in this TB failed and the TB was not further considered in the analysis. The magnitudes and phases of the differential pressure (Δ*P* = *P*_SV_ − *P*_ST_) normalized to the velocity of the SFP (*V*_SFP_) (Figure 7) were almost frequency independent. Across all TBs the magnitudes varied ≤21 dB, except in TB16 at 2525–3175 Hz where a notch was present. The phases were near 0° at frequencies ≤2 kHz and varied between −180° and +180° at higher frequencies.

## 4. Discussion

### 4.1. Handling and Limitations of the Off-the-Shelf Sensor System

In our study the Samba Preclin pressure measurement system was easy to handle and worked reliably. One major limitation of the Samba Preclin 420 LP pressure sensor is the fragile front membrane that can be damaged by mechanical stress or by particles drying on the membrane [25]. Therefore, the membrane had to be handled with great care (especially during sensor insertion). Although the sensor showed a strong robustness in our study as only one sensor was destroyed, an improved design with a protection of the membrane may prevent damage. The minimum bend radius of the fiber given by the manufacturer is 10 mm [25]. For intracochlear sound pressure measurements the technical specifications of the measurement system could be optimized. By increasing the numerical resolution and by adapting the pressure range to levels relevant for sound pressure measurements the resolution limit could be improved.

### 4.2. Effect of Transducer Insertion on SFP Vibration Responses

**Figure 8:**
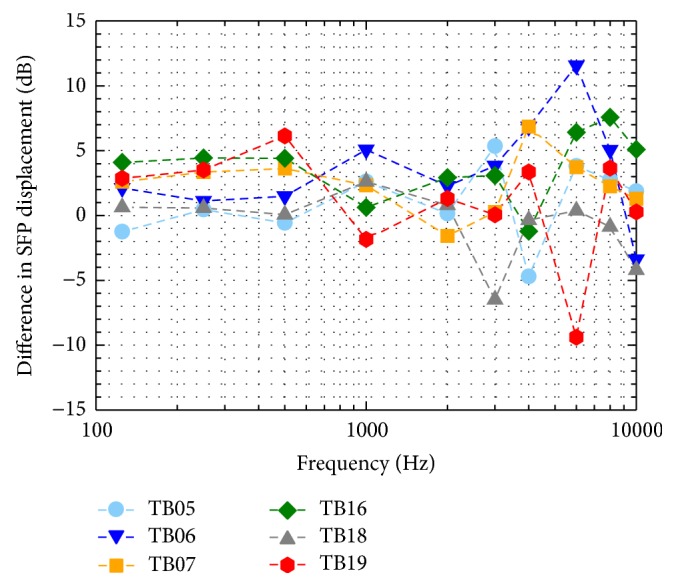
Differences in SFP vibration responses to 94 dB SPL sound stimulation at the tympanic membrane in TB preparations before and after pressure transducer insertion (Δ*d* = *d*_post_ − *d*_pre_).

After pressure transducer insertion most SFP vibration responses to sound (Figure 2) still fulfilled the modified ASTM criteria [4]. The difference between SFP vibration displacements before and after insertion of transducers (Δ*d* = *d*_post_ − *d*_pre_) was generally within 5 dB below 3 kHz and within 7 dB at higher frequencies (Figure 8). Only at 6 kHz the difference was higher in TB06 (11.6 dB) and TB19 (9.4 dB). These results indicate that the opening and reclosure of the cochlea by insertion of the pressure transducers have no pronounced effect on cochlear mechanics. This confirms the assumption that the inserted sensor membrane is much stiffer, has a much higher acoustic impedance than the round window membrane, and does not lead to major changes in natural cochlea acoustics.

### 4.3. Sealing Techniques

No correlation between the sealing material used (dental impression material in TB05–07 or silicone rubber in TB16, 18, 19) and the magnitude of *P*_SV_, *P*_ST_, and Δ*P* (Figures 3, 4, 6, and 7) was detectable. Since silicone rubber was easier to use than alginate and it was reusable in several experiments when once applied to the transducer; it is advantageous.

### 4.4. Comparison to Previous Work with Custom-Made Pressure Sensors

In the past it has been already demonstrated that the measurement of intracochlear pressure differences across the cochlear partition can be used to characterize the response levels from forward and reverse stimulation in human cadaveric TBs [6, 20–22]. The objective of this study was to investigate if intracochlear differential pressures are measurable in a similar manner with the off-the-shelf pressure transducer Samba Preclin 420 LP being originally intended for static pressure measurement. Thus, the intracochlear sound pressures *P*_SV_, *P*_ST_ and differential sound pressures Δ*P* measured here were compared (Figures 3, 4, 6, and 7) to earlier measurements [6, 21, 22] performed with custom-made sensors developed by Olson [23]. Recently [28, 29] intracochlear sound pressures were measured in scala vestibuli and scala tympani with an off-the-shelf sensor different to the one used here. In these studies a detailed comparison to results measured with custom-made sensors developed by Olson [23] was not performed. Here we used results [6, 21, 22] obtained with custom-made sensors developed by Olson [23] as a comparison criterion because this sensor type has proven to provide reliable results in the past.

Normalized to *P*_OEC_ or by *V*_SFP_, our *P*_SV_ magnitudes (Figures 3(a) and 4(a)) were largely within the minimum-maximum range of Stieger et al. [6] and Nakajima et al. [21] in the investigated frequency range. At frequencies ≥2 kHz, *P*_ST_ magnitudes (Figures 3(c) and 4(c)) were also mostly comparable to these studies but differed up to approximately 20 dB at lower frequencies. Whereas we observed a maximum variation of up to 42 dB in the magnitudes of *P*_ST_/*P*_OEC_ and up to 49 dB in the magnitudes of *P*_ST_/*V*_SFP_, the magnitudes of *P*_ST_/*P*_OEC_ in Nakajima et al. [21] and the magnitudes of *P*_ST_/*V*_SFP_ in Stieger et al. [6] varied maximally, approximately 25 dB. One potential reason for the difference between *P*_ST_ magnitudes found here and in other studies performed with a custom-made sensor [6, 21] might have been the 6.3 times (approximately 16 dB) bigger sound sensitive integration area of the Samba Preclin 420 LP pressure transducer (0.1385 mm^2^) compared to the custom-made sensor (0.0219 mm^2^). Another reason for that and for the higher variation of our *P*_ST_ magnitudes study might have been an imperfect sealing between pressure transducer and bone in our preparations. This would also explain why in our study the magnitudes of *P*_SV_/*P*_OEC_ and *P*_SV_/*V*_SFP_ were more similar (maximum variation: 20 dB and 30 dB) across the TBs than *P*_ST_ magnitudes.

In the experiments TB06, TB07, and TB16 where the magnitudes of *P*_SV_ and *P*_ST_ dropped at 8 and 10 kHz below 7 dB SNR the acoustic stimulation dropped to 70–90 dB SPL. This finding is in line with the theoretical resolution limit of the samba sensor system of 88–95 dB SPL at ≥7 kHz at the TM calculated in method, Section 2.3.

The phases of *P*_SV_ (Figure 3(b)) and *P*_ST_ (Figure 3(d)) relative to *P*_OEC_ were mostly within the range of Nakajima et al. [21]. Only at frequencies >4 kHz our *P*_ST_ phases differed significantly showing an up to 1 cycle longer delay which probably might be due to different unwrapping of the phase. Relative to *V*_SFP_, *P*_SV_ and *P*_ST_ phases (Figures 4(b) and 4(d)) were at frequencies <2 kHz comparable to Stieger et al. [6] but mostly lower at higher frequencies. A 1/2 cycle shift in *P*_ST_ phases at approximately 0.5 kHz determined by Stieger et al. [6] was not observable here. One potential reason for the lower similarity to Stieger et al. [6] might be that in their study the vibration response of the stapes was measured at the posterior crus whereas we measured it at the footplate leading to a different impact of rocking motions. However, to determine the input to the cochlea the relevant parameter is the pressure difference between SV and ST correlating to the cochlear microphonics [19]. When normalized to *P*_OEC_ (Figure 6(a)), at ≥1 kHz, the magnitude of the complex pressure difference Δ*P* = (*P*_SV_ − *P*_ST_) was within the minimum-maximum range of measurements by Nakajima et al. [21], but up to 16 dB less at lower frequencies. As mentioned before a probable reason for this discrepancy at low frequencies might have been that the sealing between pressure transducer and bone was imperfect in our experiments. On the other hand, our data was comparable in the whole frequency range (Figure 6) to two exemplary measurements of a later study [22] performed by the same researchers. This variance demonstrates that more reference data of differential intracochlear pressure measurements would be useful but is currently not available. The phases of Δ*P* relative to *P*_OEC_ were similar to Nakajima et al. [21]. When normalized to the stapes velocity, almost all magnitudes of Δ*P*/*V*_SFP_ (Figure 7(a)) were within the minimum-maximum range of Stieger et al. [6], except at frequencies <0.3 kHz where our results were maximally 15 dB less. Almost all phases of Δ*P* relative to *V*_SFP_ were within the range of Stieger et al. [6]. Only TB07 and TB16 showed at approximately 0.25 kHz (TB16) and 3 kHz (TB07 and TB16) a difference of 1/2 cycle lag.

Between 2525 and 3175 Hz where the normalized Δ*P* magnitude decreased extraordinarily in TB16 (Figures 6(a) and 7(a)), the absolute values of *P*_SV_ and *P*_ST_ were close in magnitude and phase in this experiment. Usually this might be an indication for placement of both pressure transducers accidently into the same scala. However, in this experiment the differential intracochlear pressure at all other frequencies was normal and a failure of preparation could be excluded based on the visual inspection during dissection. Hence, no explanation was found for this decrease in pressure difference in TB16.

In consideration of nonlinear effects on the normalized intracochlear pressure magnitudes, the range of acoustical stimulation levels has to be taken into account. In our study sounds were presented at 105–130 dB SPL, whereas in Stieger et al. [6] stimulation levels between 50 and 115 dB SPL and in Nakajima et al. [21] levels between 70 and 130 dB SPL were used. In Pisano et al. [22] no information about the stimulation level was provided, but it was referred to Nakajima et al. [21]. It is known that the vibration response of the stapes in human cadaveric TBs is linear with the level of acoustic stimulation up to 124 dB SPL at 0.4–6 kHz [30] and up to 130 dB SPL at 0.1–4 kHz [31]. Therefore, it can be assumed that the normalized intracochlear pressures and pressure differences measured by Stieger et al., Nakajima et al., and Pisano et al. [6, 21, 22] and our results are not subject to significant middle ear nonlinearities although our minimum stimulation levels were higher in experiments. In one experiment we stimulated acoustically first with sound pressure levels of 110–130 dB SPL and second with levels of 90–120 dB SPL. When normalized to *P*_OEC_ the magnitudes of *P*_SV_ and *P*_ST_ were similar within 3 dB except at 3175 Hz where a decrease in *P*_ST_ by 12 dB was found for the lower simulation level.

## 5. Conclusion

Intracochlear pressure differences obtained in this study with the off-the-shelf pressure transducer Samba Preclin 420 LP were comparable to results obtained with custom-made sensors [23] at frequencies of 1–10 kHz and differed up to 16 dB below 1 kHz. Additionally we could show that insertion of the pressure transducers had a minor effect of <5 dB on the stapes vibration response to sound. Our results demonstrate that the Samba Preclin 420 LP is usable for simultaneous measurements of intracochlear sound pressures in SV and ST in human cadaveric temporal bones with sufficient SNR and sensitivity.
